# Carotid Plaque Assessment Reclassifies Patients with Inflammatory Bowel Disease into Very-High Cardiovascular Risk

**DOI:** 10.3390/jcm10081671

**Published:** 2021-04-13

**Authors:** Alejandro Hernández-Camba, Marta Carrillo-Palau, Laura Ramos, Noemi Hernández Alvarez-Buylla, Inmaculada Alonso-Abreu, Anjara Hernández-Pérez, Milagros Vela, Laura Arranz, Manuel Hernández-Guerra, Miguel Ángel González-Gay, Iván Ferraz-Amaro

**Affiliations:** 1 Division of Gastroenterology, Hospital Universitario de Nuestra Señora de la Candelaria, 38010 Tenerife, Spain; dr.alejandrohc@gmail.com (A.H.-C.); milvelillas@yahoo.es (M.V.); lauraarranz@gmail.com (L.A.); 2 Division of Gastroenterology, Hospital Universitario de Canarias, 38320 Tenerife, Spain; martacarry@yahoo.es (M.C.-P.); laura7ramos@gmail.com (L.R.); noehdz78@hotmail.com (N.H.A.-B.); macuaa@hotmail.com (I.A.-A.); anjarahdez@gmail.com (A.H.-P.); mhernand@ull.edu.es (M.H.-G.); 3 Division of Rheumatology, Hospital Universitario Marqués de Valdecilla, Universidad de Cantabria, 39008 Santander, Spain; miguelaggay@hotmail.com; 4 Epidemiology, Genetics and Atherosclerosis Research Group on Systemic Inflammatory Diseases, Hospital Universitario Marqués de Valdecilla, IDIVAL, 39008 Santander, Spain; 5 Cardiovascular Pathophysiology and Genomics Research Unit, School of Physiology, Faculty of Health Sciences, University of the Witwatersrand, Johannesburg 2193, South Africa; 6 Division of Rheumatology, Hospital Universitario de Canarias, 38320 Tenerife, Spain

**Keywords:** inflammatory bowel disease, SCORE, carotid plaques, cardiovascular risk

## Abstract

The addition of carotid ultrasound into cardiovascular (CV) risk scores has been found to be effective in identifying patients with chronic inflammatory diseases at high-CV risk. We aimed to determine if its use would facilitate the reclassification of patients with inflammatory bowel disease (IBD) into the very high-CV-risk category and whether this may be related to disease features. In this cross-sectional study encompassing 186 IBD patients and 175 controls, Systematic Coronary Risk Evaluation (SCORE), disease activity measurements, and the presence of carotid plaques by ultrasonography were assessed. Reclassification was compared between patients and controls. A multivariable regression analysis was performed to evaluate if the risk of reclassification could be explained by disease-related features and to assess the influence of traditional CV risk factors on this reclassification. After evaluation of carotid ultrasound, a significantly higher frequency of reclassification was found in patients with IBD compared to controls (35% vs. 24%, *p* = 0.030). When this analysis was performed only on subjects included in the SCORE low-CV-risk category, 21% IBD patients compared to 11% controls (*p* = 0.034) were reclassified into the very high-CV-risk category. Disease-related data, including disease activity, were not associated with reclassification after fully multivariable regression analysis. Traditional CV risk factors showed a similar influence over reclassification in patients and controls. However, LDL-cholesterol disclosed a higher effect in controls compared to patients (beta coef. 1.03 (95%CI 1.02–1.04) vs. 1.01 (95%CI 1.00–1.02), interaction *p* = 0.035) after adjustment for confounders. In conclusion, carotid plaque assessment is useful to identify high-CV risk IBD patients.

## 1. Introduction

Inflammatory bowel disease (IBD), which includes both Crohn’s disease (CD) and ulcerative colitis (UC), is characterized by chronic inflammation of the gastrointestinal tract. There is growing evidence that IBD patients have a higher incidence of cardiovascular (CV) events compared to the general population [1,2,3,4]. This occurs despite having a lower burden of traditional CV risk factors [5]. Systemic inflammation load may play an important role in the process of accelerated atherosclerosis in these patients [6]. It leads to increased oxidative stress and elevated levels of inflammatory cytokines, such as tumor necrosis factor-α (TNF-α), yielding phenotypic changes in smooth muscle cells and promoting the development of atherosclerosis and CV disease in IBD patients [7]. In addition to the endogenous factors and cytokines, it has been suggested that due to the compromised intestinal mucosal barrier, endotoxins, and bacterial lipopolysaccharides produced by intestinal microflora can enter into circulation and activate inflammatory responses that lead to atherosclerosis [8].

Predictive scoring algorithms for CV disease, such as the European Systematic Coronary Risk Assessment (SCORE), have been found to underestimate the actual CV risk of patients with chronic inflammatory diseases [9,10]. In this sense, the 2016 European Guidelines for the Prevention of Cardiovascular Diseases in Clinical Practice proposed the evaluation of the plaque of the carotid artery by ultrasound as a practical way to reclassify those people for whom the SCORE may underestimate their actual CV risk [11]. This is because the presence of carotid plaque is considered an excellent tool to identify high-CV risk in patients included in the SCORE category of moderate CV risk. This is due to the fact that the assessment of carotid plaque burden with ultrasound has been demonstrated to be predictive of CV events because peripheral arterial disease represents an independent risk factor for CV death. For this reason, reclassifying the CV risk category, and identifying patients at a very high risk, would allow more intense preventive measures to be taken. In this regard, previous studies on rheumatoid arthritis, the prototype of chronic inflammatory disease, demonstrated that patients categorized as moderate-CV risk according to the SCORE often present with carotid plaques and, consequently, they should be included in the very high-CV risk category [12,13].

To the best of our knowledge, there is no extensive information on the predictive value of risk charts algorithms, in particular the SCORE, to identify IBD patients at high-CV risk [14,15]. This is also the case for the possible relevance of the presence of carotid plaques to reclassify patients with IBD as having high-CV risk. Taking together all these considerations, in the present study, we aimed to determine how the carotid ultrasound assessment may help identify IBD patients who were included in the categories of low and intermediate-CV risk when the SCORE was applied. We additionally assessed if the reclassification into the very-high CV risk due to the presence of carotid plaques may be influenced by disease characteristics, in particular by the activity of the disease.

## 2. Materials and Methods

### 2.1. Study Participants

The cross-sectional study included 186 consecutive patients with IBD and 175 controls. All of them were 18 years old or older and had a clinical diagnosis of IBD based upon clinical, endoscopic, and histological criteria at least within the previous 12 months. They had been diagnosed by gastroenterologists and were periodically followed-up at the Gastroenterology Outpatient Clinics of Hospital Universitario de Canarias and Hospital Universitario Nuestra Señora de La Candelaria. For the purpose of inclusion in the present study, IBD disease duration had to be ≥1 year. Although long-term anti-TNF-α therapy has been associated with a decreased risk of acute arterial events in patients with IBD [7], those undergoing anti-TNF-α, or other biological therapies, were not excluded from the present study. Likewise, since glucocorticoids are used in the management of IBD, patients taking prednisone were not excluded. The controls were community-based, recruited by general practitioners in primary health centers. Controls with family history of any inflammatory bowel disease or other autoimmune disorder were excluded. None of the patients and controls had established CV disease such as coronary heart disease (angina or myocardial infarction) or heart failure, strokes or transient ischemic attack, peripheral arterial disease, and aortic disease such as aortic aneurysm. Since diabetes mellitus is considered an equivalent of very-high CV risk, diabetic patients and controls were also excluded. The study protocol was approved by the Institutional Review Committee at Hospital Universitario de Canarias and Hospital Universitario Nuestra Señora de La Candelaria, both in Spain, and all subjects provided informed written consent (approval no. CHUC_2019_103). Research carried out with human subjects was in compliance with the Helsinki Declaration.

### 2.2. Assessments and Data Collection

Surveys in IBD patients and controls were performed to assess CV risk factors and medication. Hypertension was defined as a systolic or a diastolic blood pressure higher than 140 and 90 mmHg, respectively. Dyslipidemia was defined if one of the following factors was present: total cholesterol >200 mg/dL, triglycerides >150 mg/dL, HDL-cholesterol <40 mg/dL in men or <50 mg/dL in women, or LDL-cholesterol >130 mg/dL. Standard techniques were used to measure serum lipids, high-sensitivity C-reactive protein (CRP), and fecal calprotectin. Additionally, it was registered if the patient had a recent colonoscopy or magnetic resonance enterography. Disease activity in CD was assessed through Crohn’s Disease Activity Index (CDAI) and the Harvey-Bradshaw Index (HBI) [16]. CDAI was broken down into asymptomatic remission (0 to 149 points), mildly to moderately active (150 to 220), moderately to severely active (221 to 450 points), and severely active to fulminant disease (451 to 1100 points) categories as previously described [17]. Similarly, the Harvey–Bradshaw Index was categorized as remission (0 to 4 points), mildly active disease (5 to 7 points), moderately active disease (8 to 16 points), and severely active disease (17 to 100 points) [16]. Disease activity in UC was calculated through the partial Mayo Clinic score [18]. In patients with IBD, physical activity was assessed through the International Physical Activity Questionnaire (IPAQ) short form, and data was presented as metabolic equivalent-of-task (MET)-minutes per week [19]. All data acquisition regarding fecal calprotectin assessment and questionnaires evaluation, including carotid ultrasound, were performed in the same visit day.

### 2.3. Carotid Ultrasound Assessment

Carotid ultrasound was performed to determine carotid intima media thickness (cIMT) in the common carotid artery and to detect focal plaques in the extracranial carotid tree both in patients with IBD and in controls [12,13]. A commercially available scanner, Mylab 70, (Esaote, Genoa, Italy) equipped with a 7–12 MHz linear transducer and an automated software-guided radiofrequency technique—Quality Intima Media Thickness in real-time (QIMT, Esaote, Maastricht, Holland)—was used for this purpose. Based on the Mannheim consensus, plaque criteria in the accessible extracranial carotid tree (common carotid artery, bulb, and internal carotid artery) were defined as follows: a focal protrusion in the lumen measuring at least cIMT >1.5 mm; a protrusion at least 50% greater than the surrounding cIMT; or an arterial lumen encroaching >0.5 mm [20].

### 2.4. Statistical Analysis

Patients and controls with carotid plaques based on ultrasound assessment were reclassified into very-high CV risk category. Subjects without plaques were maintained in their original SCORE category. cIMT was not used to determine reclassification because according to current guidelines [11], cIMT is not considered an unequivocal CV disease on imaging. Demographic and clinical characteristics were shown as frequencies for binary variables. Continuous variables data were expressed as mean ± standard deviation (SD) or as a median and interquartile range (IQR) for non-normally distributed variables. Univariable differences between patients and controls were assessed through Student’s *t*-test, U Mann–Whitney, Chi squared, or Fisher Exact tests according to normal distribution or the number of subjects. Logistic regression analysis adjusted for the variables with a *p*-value below 0.20 in the univariate analysis was performed to assess the relation of IBD disease-related data with the presence of reclassification. Interaction factors were added to the regression models when we addressed the comparison of the effect (beta coefficients) between controls and IBD patients. All of the analyses used a 5% two-sided significance level and were performed using SPSS software, v. 25 (IBM, Chicago, IL, USA) and STATA software, v.15/SE (Stata Corp., College Station, TX, USA). A *p*-value < 0.05 was considered statistically significant.

## 3. Results

### 3.1. Demographic, Laboratory, and Disease-Related Data

A total of 186 IBD patients and 175 sex-matched controls with a mean ± SD age of 45 ± 12 and 48 ± 10 years, respectively, were included in this study. Demographic and disease-related characteristics of the participants are shown in Table 1. Body mass index (27 ± 5 vs. 26 ± 4 kg/m^2^, *p* = 0.008) and waist circumference (93 ± 12 vs. 90 ± 14 cm, *p* = 0.048) were higher in IBD patients than controls. In this regard, whereas there were not differences in the prevalence of smoking or hypertension, patients with IBD were more commonly obese (27% vs. 13%, *p* = 0.001) and more frequently met the definition for dyslipidemia (73% vs. 56%, *p* = 0.017).

The median disease duration of IBD was 12 years (IQR 8–19). CD patients had mostly the ileal and non-stricturing, non-penetrating, types. Median CDAI score was 39 (IQR 7–85), and 89% of the patients were considered to be in the asymptomatic remission category. Similarly, the Harvey–Bradshay Index was 2 (IQR 0–4), and most of the patients (81%) were in the remission category of this index. Regarding UC, 49% were pancolitis, and 76% of the patients had a partial Mayo score inferior to 2 points. Additional information regarding disease-related data is shown in Table 1.

Concerning carotid ultrasound assessment, 33% of the IBD patients had carotid plaques compared to 25% of controls (*p* = 0.067). The average cIMT in patients and controls was 641 ± 137 mm and 604 ± 115 mm, respectively (*p* = 0.006).

### 3.2. SCORE Risk Category Reclassification after Carotid Sonography

Following SCORE risk chart stratification, 124 (67%) patients and 133 (76%) controls were included in the low-CV risk category. Only 2 controls and 1 patient fulfilled the definition for very high-CV risk when the risk charts were applied (Table 2). Interestingly, carotid ultrasound assessments disclosed a significantly higher frequency of reclassification in IBD patients compared to controls (34% vs. 24%, *p* = 0.030). In this regard, 26 of the 124 patients (21%) and 15 of the 133 controls (11%) who met the definition of low CV risk, according to the SCORE risk tables, had carotid plaques; consequently, they were reclassified in the very high-risk category (21% vs. 11%, *p* = 0.034). Thirty of 55 patients (55%) and 17 of 28 controls (61%) (*p* = 0.59) included in the moderate-CV risk SCORE category had carotid plaques and were also reclassified into the very high-CV risk category. Similarly, 8 of 11 IBD patients (73%) and 6 of 6 controls (100%) included in the high-CV risk SCORE category prior to carotid ultrasound assessment were reclassified into the very high-risk category once that this test was performed (*p* = 0.52) (Table 2).

### 3.3. Differences in the Effect of Traditional CV Risk Factors on Reclassification in Controls and IBD Patients

Most of the demographics, traditional CV risk factors, and lipid profile-related molecules were associated with reclassification in both patients and controls (Table 3). However, the magnitude of these relations differed between patients and controls. In this sense, the beta coefficients of male gender, body mass index (BMI), systolic blood pressure, and dyslipidemia, in their relation to reclassification, were higher in controls when compared to patients with IBD. However, when differences between populations were assessed through the addition of interaction factors into the regression model, beta coefficients were not found to be different.

Almost all the lipid profile related-molecules were associated with reclassification in both patients and controls. In general, beta coefficients were higher in controls compared to patients, but statistical significance was not reached. Only the effect of LDL-cholesterol on reclassification was found to be greater in controls compared to IBD patients (beta coef. 1.03 (95%CI 1.02–1.04) vs. 1.01 (95%CI 1.00–1.02), interaction *p* = 0.035) after multivariable analysis adjusting for age and sex (Table 3).

### 3.4. IBD-Related Features Association with Reclassification

Disease-related features were not related to reclassification (Table 4). In this sense, neither disease duration, disease activity scores, laboratory data such as calprotectin, nor the different treatments used were not associated with reclassification. Only the onset of CD after 40 years and the use of methotrexate were significantly associated with reclassification in the univariable analysis. However, after adjustment in a fully multivariable analysis, these relationships were lost.

## 4. Discussion

Patients with IBD have a higher risk of atherosclerosis and, consequently, a higher risk of CV events. For this reason, the identification of IBD patients at high-CV risk is crucial. Our work is the first study that includes the use of carotid ultrasound to reclassify the CV risk of IBD patients. According to our results, the effect of the detection of carotid plaques by carotid ultrasound, which allows the reclassification of individuals in the category of very high CV risk, is observed more frequently in patients with IBD than in controls.

Carotid ultrasound has been previously used for the reclassification of the CV risk of patients with chronic inflammatory diseases. In this regard, in an earlier work of our group that included 343 patients diagnosed with ankylosing spondylitis and 177 controls, patients were more likely reclassified into the very high-CV risk category than controls following carotid ultrasound assessment [21]. In addition, patients with psoriatic arthritis were more frequently reclassified into very high-CV risk following carotid ultrasound assessment than controls [22]. In these patients with psoriatic arthritis, the reclassification was independently explained by the disease activity. Likewise, in a cross-sectional study that included 276 patients with systemic lupus erythematosus, following carotid ultrasound assessment, 32% of the patients were reclassified into the very high-CV risk category [10]. In systemic lupus erythematosus patients, disease duration and damage were independently associated with a higher risk of reclassification. The findings shown in the present study go in the same direction and confirm that carotid ultrasound is useful to identify IBD patients at high risk of CV disease.

IBD-related features have been associated with CV risk. For example, in a nationwide population-based cohort of 28,833 individuals diagnosed with IBD compared with IBD-free individuals, a markedly increased risk of ischemic heart disease was seen within the first year after IBD diagnosis [23]. The risk of ischemic heart disease was lower among patients with IBD using 5-aminosalicylic acid than among non-users, in particular in the group of oral glucocorticoid users, which was used as a proxy for disease severity. Likewise, patients treated surgically or with thiopurines and TNF alpha inhibitors tended to have reduced incidence rate ratio for ischemic heart disease. In another report, patients exposed to anti-TNF therapy compared to those not exposed, but not to thiopurines, were associated with a lower risk of acute arterial events [7]. The cross-sectional nature of our study and the fact that most of our patients were in clinical remission at the time of the assessment may explain why IBD-related characteristics did not show an effect on CV risk reclassification. Perhaps, mechanisms related to the disease itself that are not captured by the clinical manifestations that we registered are responsible for this greater reclassification. However, IBD follows a natural course with alternating periods of remission and relapse. This reinforces the claim that the disease itself, characterized by a chronic pro-inflammatory state, even in the latent stages of the disease, may explain a higher risk of CV reclassification, due to a greater risk of severe atherosclerotic disease in these patients. The fact that in our study, some traditional CV risk factors, such as the presence of dyslipidemia and the body mass index, had a greater effect in controls than in patients suggests that chronic inflammation may be the main mechanism that leads to an increased risk of reclassification in patients with IBD. However, we cannot exclude that a genetic component may also contribute to increased risk of CV disease in IBD, as has been described in other chronic inflammatory diseases [24,25].

In a recent report of the European Society of Hypertension Working Group on Vascular Structure and Function and the ARTERY Society (Association for Research into Arterial Structure, Physiology) evidence regarding the vascular consequences of inflammation has been extensively reviewed [8]. In this statement, it has been demonstrated that immune-mediated mechanisms related to inflammation influence arterial physiology and lead to vascular dysfunction such as atherosclerosis and arterial stiffening. Moreover, it is shown that chronic inflammatory diseases such as rheumatoid arthritis, IBD, and psoriasis are accompanied by profound arterial dysfunction, which is proportional to the severity of inflammation. Taking this into account, we believe that our findings regarding a higher reclassification into the very-high CV risk category after carotid ultrasound assessment in patients with IBD may be driven by the inflammation present in this disease.

As mentioned above, the cross-sectional nature of the present study is a limitation that does not allow us to know if patients with IBD in whom their risk was reclassified will develop CV events. However, it was recently confirmed that reclassification into very high-CV risk by identifying carotid plaques after carotid ultrasound [12,13] is useful as the best predictor of future CV events in prospectively followed-up patients with rheumatoid arthritis. Another possible limitation may be that the patients and controls were not the same age. However, the size effect of this difference was small (3 years). In this regard, it should be noted that SCORE risk values can be compared between populations of different ages because SCORE is already weighted by age. Furthermore, all the multivariable analyses performed in the present study were adjusted for age.

Finally, in our study, only 34 patients (18%) had arthritis involvement in the form of spondyloarthritis or other types of arthritis associated with IBD. This small number of patients with arthritis constituted another limitation that precluded a comparison of CV risk between them and those without arthritis.

In conclusion, the use of carotid ultrasound in patients with IBD allows identifying IBD patients at very high risk of CV disease. Due to this, we propose the use of carotid ultrasound as an additional tool for the identification of subclinical atherosclerosis in these patients.

## Figures and Tables

**Table 1:** Characteristics of patients with inflammatory bowel disease (IBD) and controls.

IBD Patients	Controls (*n* = 175)	IBD Patients (*n* = 186)	*p*-Value
Age, years	45 ± 12	48 ± 10	**0.009**
Male, *n* (%)	89 (51)	85 (46)	0.30
Body mass index, kg/m^2^	26 ± 4	27 ± 5	**0.008**
Abdominal circumference, cm	90 ± 14	93 ± 12	0.066
Systolic blood pressure, mmHg	125 ± 15	125 ± 19	0.83
Diastolic blood pressure, mmHg	78 ± 9	74 ± 12	**0.000**
Cardiovascular co-morbidity			
Current smokers, *n* (%)	32 (18)	36 (19)	0.84
Hypertension, *n* (%)	20 (11)	31 (17)	0.16
Dyslipidemia, *n* (%)	98 (56)	136 (73)	**0.004**
Obesity, *n* (%)	22 (13)	50 (27)	**0.001**
Laboratory and lipid profile			
CRP, mg/L	0.8 (0.5–2.0)	1.8 (0.9–3.6)	**0.030**
Cholesterol, mg/dL	200 ± 34	204 ± 49	0.37
Triglycerides, mg/dL	103 ± 55	147 ± 88	**0.000**
HDL-cholesterol, mg/dL	58 ± 17	57 ± 18	0.53
LDL-cholesterol, mg/dL	120 ± 31	117 ± 40	0.44
LDL:HDL cholesterol ratio	2.22 ± 0.87	2.17 ± 0.86	0.60
Non-HDL cholesterol, mg/dL	142 ± 36	146 ± 43	0.25
Atherogenic index	3.64 ± 1.06	3.77 ± 1.16	0.27
IBD related data			
Crohn’s disease, *n* (%)		127 (68)	
Ulcerative colitis, *n* (%)		59 (32)	
Disease duration since diagnosis, years		12 (8–19)	
Crohn’s Disease related data, *n* (%)			
A1 below 16 years		19 (15)	
A2 between 17 and 40 years		79 (62)	
A3 above 40 years		55 (43)	
L1 ileal		55 (43)	
L2 colonic		23 (18)	
L3 ileocolonic		49 (39)	
L4 isolated upper disease		11 (9)	
B1 non-stricturing, non-penetrating		71 (56)	
B2 stricturing		45 (35)	
B3 penetrating		14 (11)	
CDAI score		39 (7–85)	
Asymptomatic remission		113 (90)	
Mildly to moderately active CD		10 (8)	
Moderately to severely active CD		3 (2)	
Severely active to fulminant disease		0 (0)	
Harvey-Bradshaw Index		2 (0–4)	
Clinical remission		103 (82)	
Mildly active disease		14 (11)	
Moderately active disease		8 (6)	
Severely active disease		1 (1)	
Ulcerative Colitis related data, *n* (%)			
Proctosigmoiditis		6 (10)	
Left-sided colitis		22 (37)	
Pancolitis		29 (49)	
Partial Mayo score		1 (0–1)	
<2		45 (76)	
≥2		14 (24)	
Fecal calprotectin, mcg/g			
<120		58 (31)	
≥120		68 (37)	
Perianal disease, *n* (%)		22 (12)	
Previous surgery, *n* (%)		54 (29)	
Extraintestinal manifestations		53 (28)	
Arthritis, *n* (%)		34 (18)	
Uveitis, *n* (%)		4 (2)	
Erythema nodosum, *n* (%)		4 (2)	
Psoriasis, *n* (%)		5 (3)	
Current prednisone, *n* (%)		6 (3)	
Prednisone, mg/day		8 (5–20)	
Oral Mesalazine, *n* (%)		60 (32)	
Methotrexate, *n* (%)		21 (11)	
Azathioprine, *n* (%)		58 (31)	
Anti-TNF therapy, *n* (%)		56 (30)	
Adalimumab, n (%)		23 (12)	
Infliximab, n (%)		33 (18)	
Ustekinumab, *n* (%)		8 (4)	
Vedolizumab, *n* (%)		5 (3)	
Tofacitinib, *n* (%)		4 (2)	
Certolizumab, *n* (%)		1 (1)	
Carotid intima media assessment			
Carotid plaque, *n* (%)	43 (25)	62 (33)	0.067
bilateral, *n* (%)	19 (11)	30 (16)	0.14
cIMT, microns	604 ± 115	641 ± 137	**0.006**

Data represent means ± SD or median (interquartile range) when data were not normally distributed. BMI: body mass index; CRP: C reactive protein; LDL: low-density lipoprotein. HDL: high-density lipoprotein; TNF: tumor necrosis factor; cIMT: carotid intima media. CDAI: Crohn’s Disease Activity Index. Dyslipidemia was defined if one of the following was present: total cholesterol > 200 mg/dL, triglycerides > 150 mg/dL, HDL-cholesterol < 40 in men or <50 mg/dL in women, or LDL-cholesterol > 130 mg/dL. CDAI was categorized as 0 to 149: Asymptomatic remission; 150 to 220 points: Mildly to moderately active; 221 to 450 points: Moderately to severely active; 451 to 1100 points: Severely active to fulminant disease. Harvey–Bradshaw Index was categorized as 0 to 4 points Clinical remission; 5 to 7 points: Mildly active disease; 8 to 16 points: Moderately active disease; 17 to 100 points: Severely active disease. Extraintestinal manifestations refer to those related to musculoskeletal, dermatological or ocular systems. Significant *p*-values are depicted in bold.

**Table 2:** Systematic Coronary Risk Evaluation (SCORE) risk category reclassification after carotid ultrasound assessment in patients with inflammatory bowel disease (IBD) and controls.

Initial Score Risk Category		Risk Category after Carotid Ultrasound Assessment				% Patients Reclassified	*p*
		Low	Moderate	High	Very High		
Controls							
Low	133	118			15	11%	
Moderate	28		11		17	61%	
High	11			3	8	73%	
Very High	2				2	-	
Total	174	118	11	3	42	24%	
IBD patients							
Low	124	98			26	21%	**0.034**
Moderate	55		25		30	55%	0.59
High	6			0	6	100%	0.52
Very High	1				1	-	-
Total	186	98	25	0	63	34%	**0.030**

SCORE: Systematic Coronary Risk Evaluation; IBD: inflammatory bowel disease. *P*-values in every risk category represent the comparison between patients and controls for that category. Significant *p*-values are given in bold.

**Table 3:** Differences in the effect of traditional cardiovascular risk factors on reclassification in IBD patients and controls.

IBD Patients	Reclassified after Carotid Ultrasound			
	OR (95% CI), *p*		Interaction	
	Controls	IBD	Univariable	Adjusted
Demographics				
Age, years	**1.13 (1.08–1.18), 0.000**	**1.13 (1.08–1.18), 0.000**	0.94	
Male, *n* (%)	**2.79 (1.31–5.95), 0.008**	1.58 (0.85–2.91), 0.15	0.25	
Body mass index, kg/m^2^	**1.12 (1.03–1.21), 0.010**	1.02 (0.96–1.08), 0.59	0.078	0.79
Abdominal circumference, cm	**1.05 (1.02–1.08), 0.001**	**1.03 (1.00–1.06), 0.034**	0.29	
Systolic blood pressure, mmHg	**1.04 (1.01–1.06), 0.003**	**1.03 (1.01–1.04), 0.005**	0.44	
Diastolic blood pressure, mmHg	1.04 (1.00–1.08), 0.065	1.02 (1.00–1.05), 0.087	0.58	
Cardiovascular co-morbidity				
Smoking, *n* (%)	1.13 (0.46–2.75), 0.80	1.57 (0.74–3.30), 0.24	0.58	
Hypertension, *n* (%)	2.52 (0.95–6.69), 0.063	**2.97 (1.35–6.53), 0.007**	0.80	
Dyslipidemia, *n* (%)	**2.47 (1.11–5.50), 0.026**	2.01 (0.94–4.28), 0.071	0.71	
Obesity, *n* (%)	0.72 (0.23–2.25), 0.57	1.04 (0.53–2.07), 0.91	0.58	
Laboratory and lipid profile				
CRP, mg/L	0.96 (0.85–1.09), 0.55	0.99 (0.92–1.06), 0.77	0.71	
Cholesterol, mg/dL	**1.02 (1.01–1.03), 0.001**	**1.01 (1.00–1.01), 0.035**	**0.044**	0.086
Triglycerides, mg/dL	**1.01 (1.00–1.01), 0.020**	**1.00 (1.00–1.01), 0.013**	0.43	
HDL cholesterol, mg/dL	**0.97 (0.95–0.99), 0.044**	0.99 (0.97–1.01), 0.29	0.30	
LDL cholesterol, mg/dL	**1.03 (1.02–1.04), 0.000**	1.01 (1.00–1.02), 0.057	**0.009**	**0.035**
LDL:HDL cholesterol ratio	**2.62 (1.62–4.18), 0.000**	**1.91 (1.31–2.79), 0.001**	0.32	
Non-HDL cholesterol, mg/dL	**1.02 (1.01–1.04), 0.000**	**1.01 (1.00–1.02), 0.005**	0.059	0.21
Atherogenic index	**2.00 (1.38–2.90), 0.000**	**1.61 (1.21–2.14), 0.001**	0.36	

Reclassification is considered the dependent variable in the logistic regression analysis. Interaction is adjusted for age and sex. Significant *p*-values are given in bold. BMI: body mass index; CRP: C reactive protein; LDL: low-density lipoprotein. HDL: high-density lipoprotein; TNF: tumor necrosis factor; cIMT: carotid intima media. Dyslipidemia was defined if one of the following was present: total cholesterol > 200 mg/dL, triglycerides > 150 mg/dL, HDL-cholesterol < 40 in men or <50 mg/dL in women, or LDL-cholesterol > 130 mg/dL.

**Table 4:** Disease-related data association with reclassification.

IBD Patients	Reclassified after Carotid Ultrasound	
	OR (95%CI), *p*	
	Unadjusted	Adjusted *
Disease duration since diagnosis, years	1.02 (0.99–1.05), 0.31	
log METs/week	1.28 (0.91–1.80), 0.15	1.20 (0.86–1.97), 0.21
Crohn’s Disease related data		
A1 below 16 years	0.45 (0.14–1.45), 0.18	0.99 (0.26–3.75), 0.99
A2 between 17 and 40 years	0.81 (0.39–1.68), 0.57	
A3 above 40 years	**2.70 (1.12–6.48), 0.026**	0.87 (0.27–2.75), 0.81
L1 ileal	0.94 (0.45–1.95), 0.86	
L2 colonic	0.97 (0.38–2.49), 0.95	
L3 ileocolonic	1.10 (0.53–2.30), 0.80	
L4 isolated upper disease	0.66 (0.17–2.63), 0.56	
B1 non-stricturing, non-penetrating	0.96 (0.46–1.98), 0.91	
B2 stricturing	0.87 (0.40–1.86), 0.71	
B3 penetrating	0.70 (0.21–2.38), 0.57	
CDAI score	1.00 (0.99–1.00), 0.40	
Asymptomatic remission	-	
Mildly to moderately active	0.42 (0.09–2.09), 0.29	
Moderately to severely active	-	
Severely active to fulminant disease	-	
log Harvey score	0.78 (0.49–1.25), 0.30	
Clinical remission	-	
Mildly active disease	0.45 (0.12–1.71), 0.24	
Moderately active disease	0.55 (0.11–2.86), 0.48	
Severely active disease	-	
Ulcerative colitis-related data		
Proctosigmoiditis	1.23 (0.20–7.46), 0.82	
Left-sided colitis	1.21 (0.38–3.86), 0.74	
Pancolitis	0.61 (0.19–1.90), 0.39	
log Partial Mayo score	0.94 (0.37–2.39), 0.90	
<2	-	
≥2	1.64 (0.50–5.43), 0.41	
Fecal calprotectin, mcg/g		
Perianal disease	0.93 (0.36–2.40), 0.87	
Previous surgery	1.41 (0.73–2.73), 0.31	
Extraintestinal manifestations	1.27 (0.64–2.51), 0.50	
Arthritis	1.07 (0.49–2.32), 0.86	
Uveitis	1.97 (0.27–14.30), 0.50	
Erythema nodosum	-	
Psoriasis	0.48 (0.05–4.36), 0.51	
Current prednisone	0.39 (0.50–3.41), 0.40	
Prednisone, mg/day	-	
Oral mesalazine	1.12 (0.58–2.14), 0.74	
Methotrexate	**3.07 (1.22–7.74), 0.018**	1.71 (0.55–5.33), 0.36
Azathioprine	0.86 (0.44–1.67), 0.66	
Anti-TNF therapy	0.73 (0.37–1.45), 0.37	
Adalimumab	0.38 (0.12–1.17), 0.093	0.48 (0.14–1.67), 0.25
Infliximab	1.18 (0.54–2.58), 0.68	
Ustekinumab	0.66 (0.13–3.35), 0.61	
Vedolizumab	1.34 (0.22–8.26), 0.75	
Tofacitinib	-	
Certolizumab	-	

Reclassification is considered the dependent variable in the logistic regression analysis. TNF: tumor necrosis factor; CDAI: Crohn’s Disease Activity Index. Significant *p*-values are given in bold. * Adjusted for age, sex, hypertension, and dyslipidemia (variables with a *p*-value < 0.20 in their relation with reclassification in Table 3).

## Data Availability

The data presented in this study are available on request to the corresponding author.
